# First titanosaur dinosaur nesting site from the Late Cretaceous of Brazil

**DOI:** 10.1038/s41598-022-09125-9

**Published:** 2022-03-24

**Authors:** Lucas E. Fiorelli, Agustín G. Martinelli, João Ismael da Silva, E. Martín Hechenleitner, Marcus Vinícius Theodoro Soares, Julian C. G. Silva Junior, José Carlos da Silva, Élbia Messias Roteli Borges, Luiz Carlos Borges Ribeiro, André Marconato, Giorgio Basilici, Thiago da Silva Marinho

**Affiliations:** 1 Centro Regional de Investigaciones Científicas y Transferencia Tecnológica de La Rioja (CRILAR-CONICET-Provincia de La Rioja-UNLaR-SEGEMAR-UNCa), Entre Ríos y Mendoza S/N, CP 5301 Anillaco, La Rioja Argentina; 2 Sección Paleontología de Vertebrados, Museo Argentino de Ciencias Naturales “Bernardino Rivadavia”-CONICET, Av. Ángel Gallardo 470, C1405DJR Buenos Aires, Argentina; 3 Centro de Pesquisas Paleontológicas L. I. Price, Complexo Cultural e Científico Peirópolis, Pró-Reitoria de Extensão Universitária, Universidade Federal do Triângulo Mineiro, Rua Estanislau Collenghi 194, Uberaba, Minas Gerais 38039-755 Brazil; 4 Fundação Cultural de Uberaba, Prefeitura Municipal de Uberaba, Praça Rui Barbosa 356, Uberaba, Minas Gerais 38010-250 Brazil; 5 Instituto de Biología de la Conservación y Paleobiología (IBICOPA), DACEFYN-CENIIT-UNLaR, Av. Luis M. de La Fuente S/N, CP 5300 Anillaco, La Rioja Argentina; 6 Department of Geology and Natural Resources, Institute of Geosciences, State University of Campinas, Rua Carlos Gomes 250, Campinas, São Paulo 13083-870 Brazil; 7 Laboratório de Paleontologia de Ribeirão Preto, Faculdade de Filosofia, Ciências e Letras de Ribeirão Preto, Universidade de São Paulo, Av. Bandeirantes, 3900, Ribeirão Preto, São Paulo 14040-901 Brazil; 8 Faculdades Associadas de Uberaba (FAZU), Fundação Educacional para o Desenvolvimento das Ciências Agrárias (FUNDAGRI), Associação Brasileira dos Criadores de Zebu (ABCZ), Av. do Tutuna, 720, Tutunas, Uberaba, Minas Gerais 38061-500 Brazil; 9 Escola Estadual Presidente João Pinheiro, Rua Menelick de Carvalho 383, Boa Vista, Uberaba, Minas Gerais 38017-070 Brazil; 10 Departamento de Geologia Sedimentar e Ambiental, Instituto de Geociências, Universidade de São Paulo, Rua Do Lago, 562, Cidade Universitária, São Paulo, 05580-080 Brazil; 11 Instituto de Ciências Exatas, Naturais e Educação (ICENE), Universidade Federal do Triângulo Mineiro (UFTM), Av. Randolfo Borges Jr. 1400, Univerdecidade, Uberaba, Minas Gerais 38064-200 Brazil

**Keywords:** Behavioural ecology, Evolutionary ecology, Palaeoecology, Palaeontology

## Abstract

Titanosaurs were successful herbivorous dinosaurs widely distributed in all continents during the Cretaceous, with the major diversity in South America. The success of titanosaurs was probably due to several physiological and ecological factors, in addition to a series of morphological traits they achieved during their evolutionary history. However, the generalist nesting behaviour using different palaeoenvironments and strategies was key to accomplish that success. Titanosaur nesting sites have been found extensively around the world, with notable records in Spain, France, Romania, India, and, especially, Argentina. Here, we describe the first titanosaur nesting site from the Late Cretaceous of Brazil that represents the most boreal nesting site for South America. Several egg-clutches, partially preserved, isolated eggs and many eggshell fragments were discovered in an Inceptisol palaeosol profile of the mining Lafarge Quarry, at the Ponte Alta District (Uberaba Municipality, Minas Gerais State), corresponding to the Serra da Galga Formation (Bauru Group, Bauru Basin). Although classical mechanical preparation and CT scans have not revealed embryonic remains in ovo, the eggs and eggshell features match those eggs containing titanosaurian embryos found worldwide. The morphology of the egg-clutches and observations of the sedimentary characteristics bolster the hypothesis that these sauropods were burrow-nester dinosaurs, as was already suggested for the group based on other nesting sites. The egg-clutches distributed in two levels along the Lafarge outcrops, together with the geopalaeontological data collected, provide clear evidence for the first colonial nesting and breeding area of titanosaur dinosaurs in Brazil.

## Introduction

Titanosaurs were a group of successful quadrupedal herbivorous sauropod dinosaurs distributed worldwide during the Cretaceous^1,2^ that achieved the largest sizes for animal on terrestrial environments^3,4^. They have a mainly Gondwanan distribution^1,5,6^, with a peak of abundance and diversity during the Late Cretaceous in South America^2,7,8^. Particularly, Brazil and Argentina, with almost 75% of the titanosaur species recorded in Patagonia^2^, are the countries that have provided most of the species and specimens, with some of the largest and smallest sauropod species known to date^3,9^. Sites around the world with a high abundance of fossil egg-clutches and eggs, in some cases in tightly packed nesting colonies, are uncommon and highlight that titanosaurs successfully reproduced in disparate environmental conditions at different latitudes^10,11^. These findings are important to acknowledge the complex and diverse reproductive biology of titanosaurs, because colony nesting entails behaviours with diverse ecological and evolutionary benefits. Thus, the colonial nesting behaviour of titanosaurs is key to understanding their success and dominance over all continents during the Cretaceous.

Titanosaur nesting sites have been found extensively around the world, with notable records in Europe and Asia^12–15^. However, the best-known titanosaur sauropod nesting sites are located in Argentina, with several prolific and remarkable localities^2,16–20^. The nesting sites from Argentina have yielded an impressive number of titanosaurian egg-clutches and even with embryo remains in ovo^2,16,17,19,21^. There is no unambiguous taxonomic certainty for these embryonic eggs, but it is likely that they belong to one of the two major groups of Late Cretaceous titanosaurs, namely Saltasauridae and Colossosauria^2^. The combination of palaeontological and sedimentological data from Argentinean sites, mainly in Patagonia and La Rioja Province, has provided crucial information on their palaeobiology, life history, physiology and embryology, reproductive strategies and nesting behaviours, as well as taphonomic and diagenetic processes associated with dinosaur eggs^11,16,18–25^. These titanosaur egg-clutches of up to 30 spherical eggs, about 12–18 cm in diameter, display a monolayered shell traditionally classified within Megaloolithidae, a parataxonomic group without any modern biological and evolutionary principles^11^.

**Figure 1:**
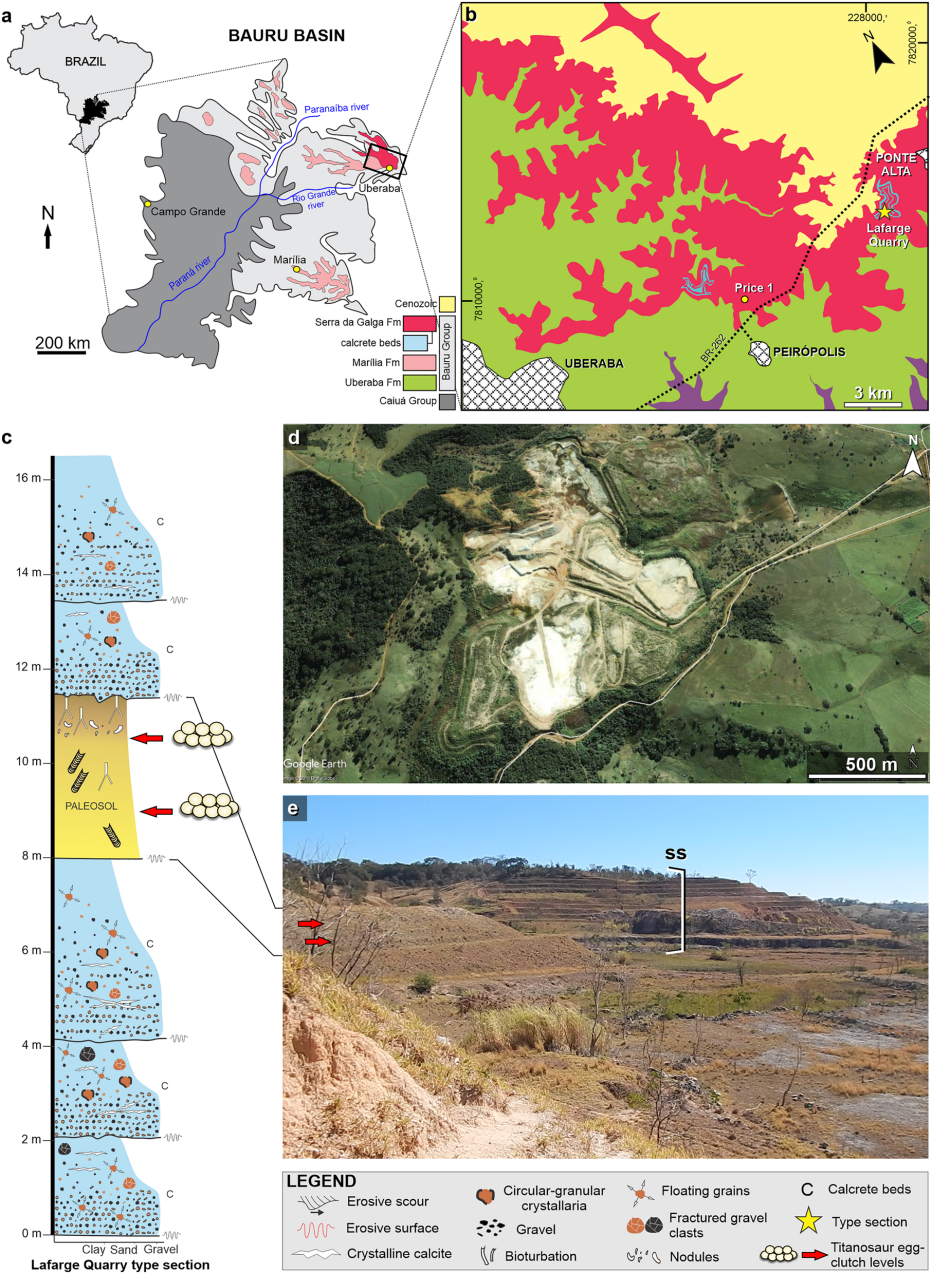
Geological map and sedimentology of Ponte Alta nesting site. (**a**) Location of the Ponte Alta site and the Serra da Galga Formation at the northeast margin of the Bauru Basin. (**b**) Lithostratigraphic map of the study area with indication of the Ponto 1 do Price and Lafarge Quarry where the Ponte Alta nesting site is located. (**c**) Stratigraphical section of the Serra da Galga Formation at the Lafarge Quarry with the provenance of titanosaur egg-clutches. (**d**) Satellite image of Lafarge Quarry in 2003; map data: Google Earth Imagen©2018 Digital Globe. (**e**) Panoramic view of the Lafarge Quarry with the outcrops of the Serra da Galga Formation, with the detail of the eggs bearing levels (arrows) and the stratigraphic section (ss). Modified from Soares et al.^32^.

Despite the extraordinary nesting sites in Argentina, records of titanosaur eggs or eggshells in other South American localities are rare, with scarce evidences in Uruguay, Brazil and Peru^26–28^. Particularly in Brazil, published oological material of titanosaurs is limited to a few isolated, incomplete eggs and eggshell fragments collected both at the “Ponto 1 do Price” site (Peirópolis) and Ponte Alta region^28–31^. However, detailed studies of titanosaur nesting sites in South America, outside of Argentina, are still lacking. Here, we report for the first time a high density of egg-clutches discovered in levels of the Serra da Galga Formation (Bauru Group)^32^ at the abandoned mining Lafarge Quarry, located in the district of Ponte Alta (Uberaba Municipality, State of Minas Gerais; Fig. 1), which represents the first confirmed titanosaur nesting site for the Late Cretaceous of Brazil and the most boreal titanosaur nesting site in South America. We describe in detail the morphological features of the egg-clutches, eggs, and eggshells, the geological and sedimentological characteristics of the egg-bearing levels, and provide palaeobiological consideration of this nesting site. The sedimentologic and palaeontologic data collected provide clear evidence for the first colonial nesting area of titanosaur dinosaurs in Brazil.

## Geological setting

The new titanosaur nesting site is located within the abandoned mining Lafarge Quarry, located about 2 km west of the rural town of Ponte Alta, Municipality of Uberaba, State of Minas Gerais, Brazil (Fig. 1a,b). This quarry is located about 35 km east of Uberaba city and 8 km east of the classic “Ponto 1 do Price” (near Peirópolis rural town)^31^. The Lafarge Quarry is a limestone mine that was worked for 26 years, reaching an extension of approximately 840,000 m^2^ (Fig. 1d,e). According to Soares et al.^32^, the Lafarge Quarry type-section is interpreted as the proximal zone of an ancient distributive fluvial system. The stratigraphic interval is mostly constituted by laterally extensive (up to 3 km) calcrete beds (2–4 m thick) that occur interbedded with few palaeosol profiles (Fig. 1c). Internally, the calcrete beds display alpha-type (i.e., abiotic origin) carbonate microstructures (e.g., circum-granullar crystallaria, crystalline calcite) that imprinted over conglomeratic and medium- to coarse-grained sandstones^32^. The calcretization is inferred to have occurred via groundwater percolation through the permeable channel deposits of the proximal zone. Conversely, the palaeosol profiles correspond to pauses in sedimentation related to the avulsion of channel belts to a distant position on the alluvial surface^32^. The duration of such breaks in fluvial sedimentation was estimated in c. 2.6–10 ky according to the degree of pedogenic carbonate cementation in palaeosols of the proximal zone (see Fig. 13 in Soares et al.^33^). The titanosaur egg-clutches and other vertebrate remains were recovered from the palaeosol interval between the two well-developed calcrete beds (Fig. 1d,e). Furthermore, the palaeosol sandy levels in the Lafarge Quarry also yielded other fossil occurrences, including fresh-water gastropods, fish (scales and teeth), crocodyliforms (teeth) and titanosaur and theropod dinosaur (teeth, bone fragments) remains, which will be described elsewhere. The Serra da Galga Formation^32^, formerly known as the Serra da Galga Member of the Marília Formation, has a taxonomically diverse tetrapod fauna including anurans (*Baurubatrachus pricei* and *Uberabatrachus carvalhoi*), fresh-water turtles (e.g., *Pricemys caiera*, *Cambaremys langertoni*), a lizard (*Pristiguana brasiliensis*), crocodilyforms (e.g., *Itasuchus jesuinoi*, *Peirosaurus torminni*, *Labidiosuchus amicum*), various theropod groups (e.g., abelisauroids, maniraptorans, birds), and titanosaurians (*Baurutitan britoi*, *Trigonosaurus pricei*, *Uberabatitan ribeiroi*)^31^ and references herein. For more information on the geology, sedimentology and palaeoecology of the region, we suggest the review papers of Martinelli and Teixeira^31^, Soares et al.^32,33^, and Martinelli et al.^34^.

## Systematic palaeontology

DINOSAURIA Owen, 1842

SAUROPODA Marsh, 1878

TITANOSAURIA Bonaparte & Coria, 1993

Titanosauria indet.

### Note

We do not follow here the classic oologic parataxonomic methodology and classification because it presents serious biological-evolutionary problems. In the section “On the methodology of egg parataxonomy” in “Methods”, we explain the problems and inconsistencies of this methodology. Here, we analyze the eggs within the context of the evolutionary trend of amniotes, in particular focused on dinosaurs.

### Material

**Figure 2:**
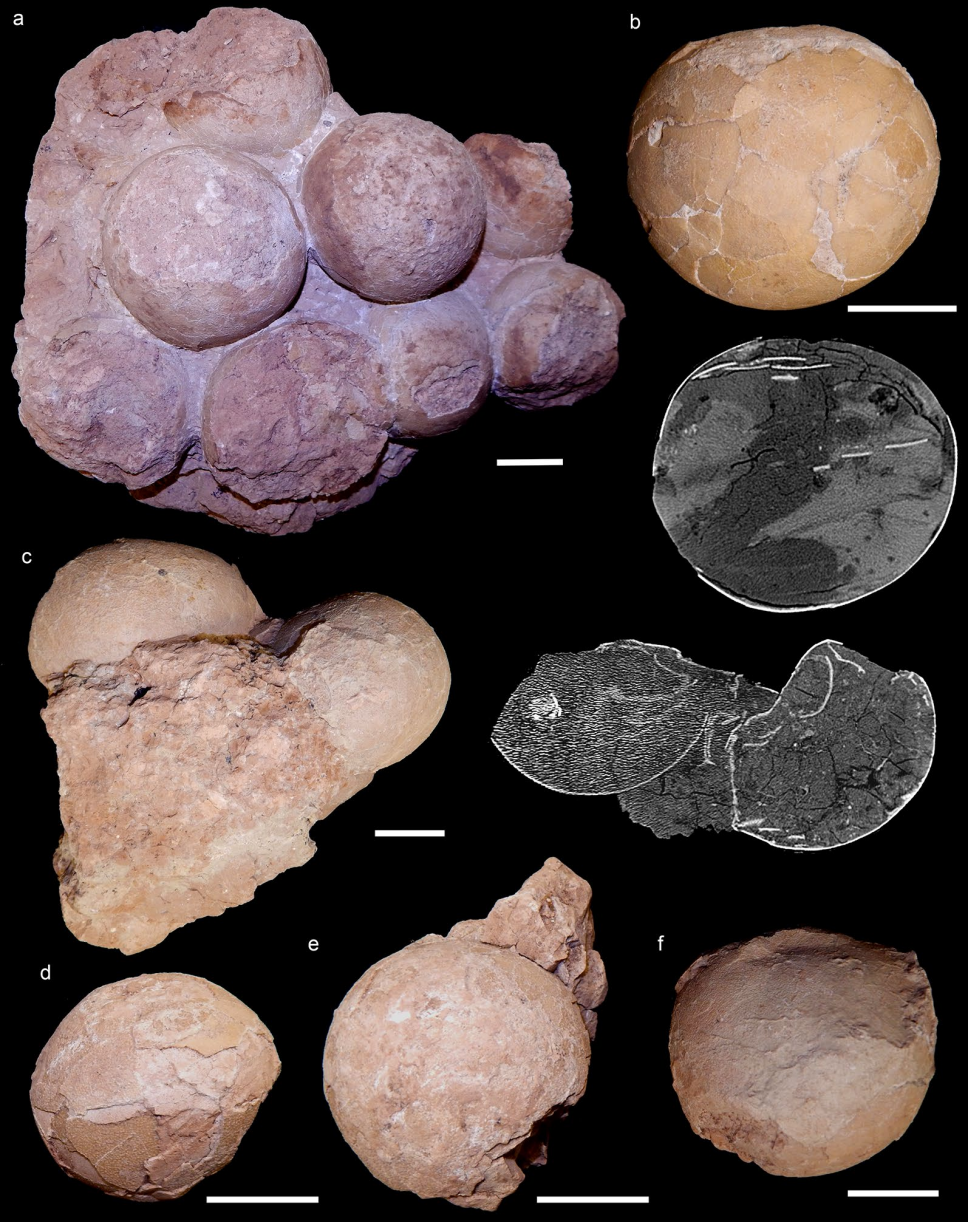
Selected titanosaurian eggs and egg-clutches collected from the Late Cretaceous Serra da Galga Formation (Bauru Group) at Ponto Alta nesting site, Uberaba Municipality, Minas Gerais State, Brazil. (**a**) CPPLIP 1798, best-preserved recovered egg-clutch, bottom view. (**b**) CPPLIP 1801, isolated egg, with accompanying tomographic slice, showing thickness of the shell and its sedimentary fill. (**c**) CPPLIP 1799, egg-clutch with accompanying tomographic slice, showing thickness of the shell, shells collapsed and its sedimentary fill. (**d**,**e**) CPPLIP 1800, two eggs found associated. (**f**) CPPLIP 1804 isolated partial egg. Scale bars 5 cm.

CPPLIP 1798, clutch with ten eggs, of which five are almost complete and spherical, all partially inserted in the sedimentary matrix (Fig. 2a); CPPLIP 1799, two spherical eggs and a fragment of a third within the sedimentary matrix (Fig. 2c); CPPLIP 1800, two almost complete and spherical eggs, with sedimentary matrix and no clear association with other specimens (Fig. 2d,e); CPPLIP 1801, an almost complete and spherical egg (Fig. 2b); CPPLIP 1802, two associated, fragmented eggs (not figured); CPPLIP 1803, three associated, fragmented eggs, two of them still within sedimentary matrix (not figured); CPPLIP 1804, two associated, almost complete eggs and one holding the sedimentary mold of the another (Fig. 2f).

### Type locality and horizon

Ponte Alta nesting site, Lafarge Quarry, Ponte Alta, Uberaba Municipality, Minas Gerais State, Brazil (Fig. 1). Sandstone beds of the Serra da Galga Formation, Bauru Group, Bauru Basin (Maastrichtian).

## The Ponte Alta nesting site

**Figure 3:**
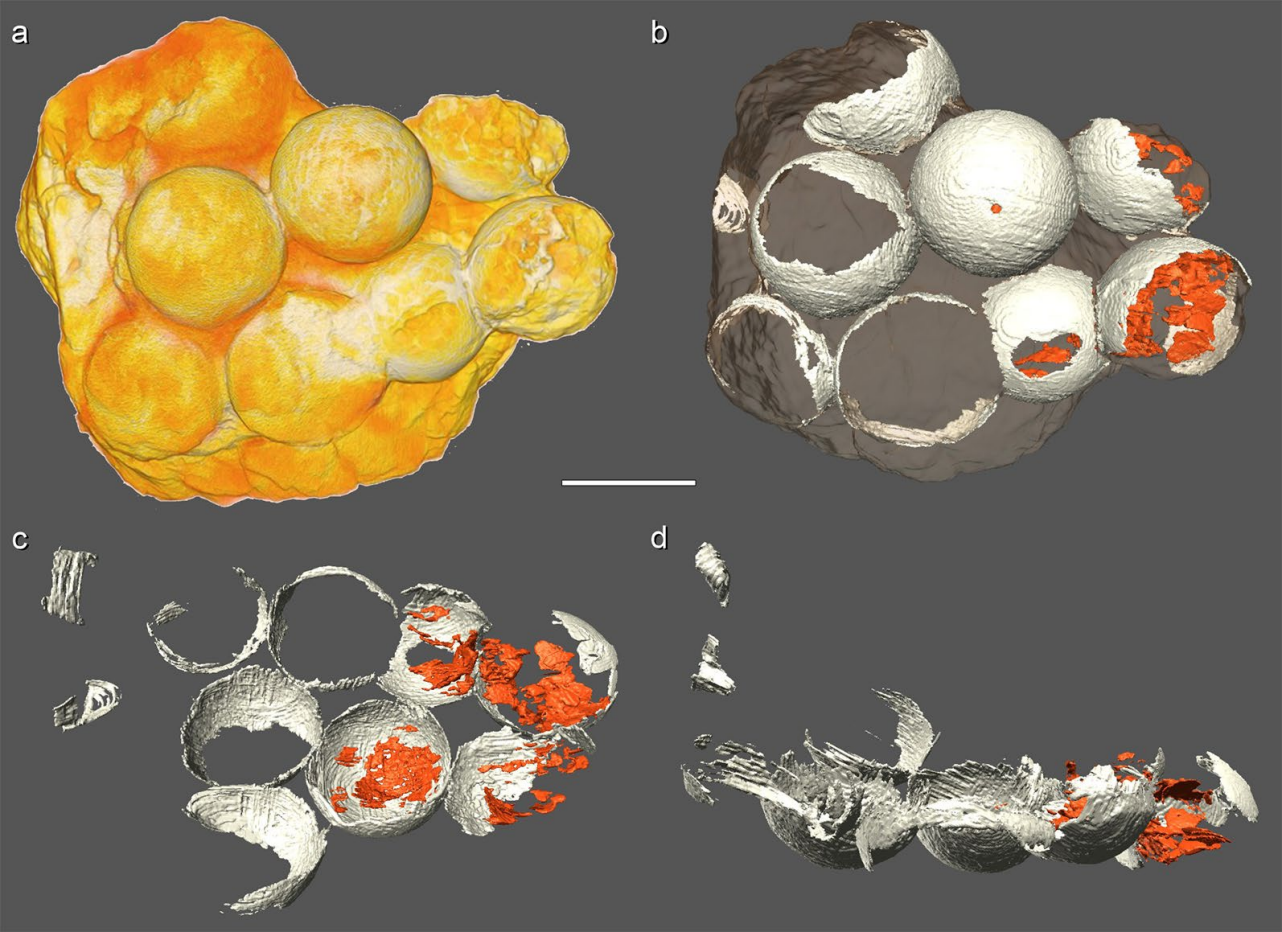
CT scans of the best-preserved egg-clutch (CPPLIP 1798). (**a**) 3D rendering of the bottom of the clutch in CPPLIP 1798. (**b**) General view of the clutch at the same orientation, showing the external part of the lower hemisphere of the eggs. (**c**) Top view of the lower egg row, showing the inner part of the lower hemisphere of the eggs. (**d**) Lateral view of CPPLIP 1798, showing lower egg row with higher shells at both ends and part of the upper egg row. In dark orange, eggshell fragments within the eggs. Note that the incompleteness (“holes”) of some eggs in B and C are due to the lack of the eggshells and/or poor resolution of the CT scan. In A the shape of each egg is maintained due to the sedimentary internal mold. Scale bar 10 cm.

The egg-clutch-bearing layers of Ponte Alta nesting site are distributed in the sandstone-palaeosol facies in between the 8 and 12 m of the stratigraphic section of the Serra da Galga Formation at the Lafarge quarry, above the lowermost exposed calcrete level (Fig. 1c–e)^32^ and the eggs have been collected within an area of approximately ~ 240 m^2^. With exception of CPPLIP 1801, all other specimens were found approximately in the same stratigraphic level (palaeosol, ~ 9 m in the stratigraphic profile), whereas the former specimen comes from almost two meters above (10.7 m) representing at least two egg-bearing levels. Although several egg-clutches were detected at the outcrop, just some were excavated and collected during the mining activities. CPPLIP 1798 is the best preserved and complete specimen of a partial clutch; it preserves a cluster of ten eggs and the clutch appears to be rounded to elliptical in shape (Fig. 2a). Of these, eight eggs can be observed in planar view, whereas two lie below them, which indicate that the clutch consisted of at least two superposed rows of eggs (Fig. 3). In addition, the eggs that occupy the periphery of the lower layer in CPPLIP 1798 are slightly higher than those in the center (Fig. 3d).

Some eggs suffered modern weathering (e.g., eroded section of eggs, breaks, wear of the surface of the shell—external surface of eggshell units), but most are quite complete. Some eggs from Ponte Alta show compressions and deformations (slightly flattened with oblate-ellipsoid-like shapes, perpendicular to clutch plane; e.g., CPPLIP 1802–1803; Fig. 2c–f), but in general they are perfectly spherical as other titanosaur eggs^2,13,17,35,36^. Most of eggs are cracked and show a partially fractured shell surface, showing other typical taphonomic alterations by sediment load (Figs. 2, 3). Because egg-surface fracturing results from sedimentary compression, we infer those titanosaurs from Ponte Alta would have buried their eggs, which is also consistent with the superposed rows of eggs (see taphonomic comments on “Discussion” section).

**Figure 4:**
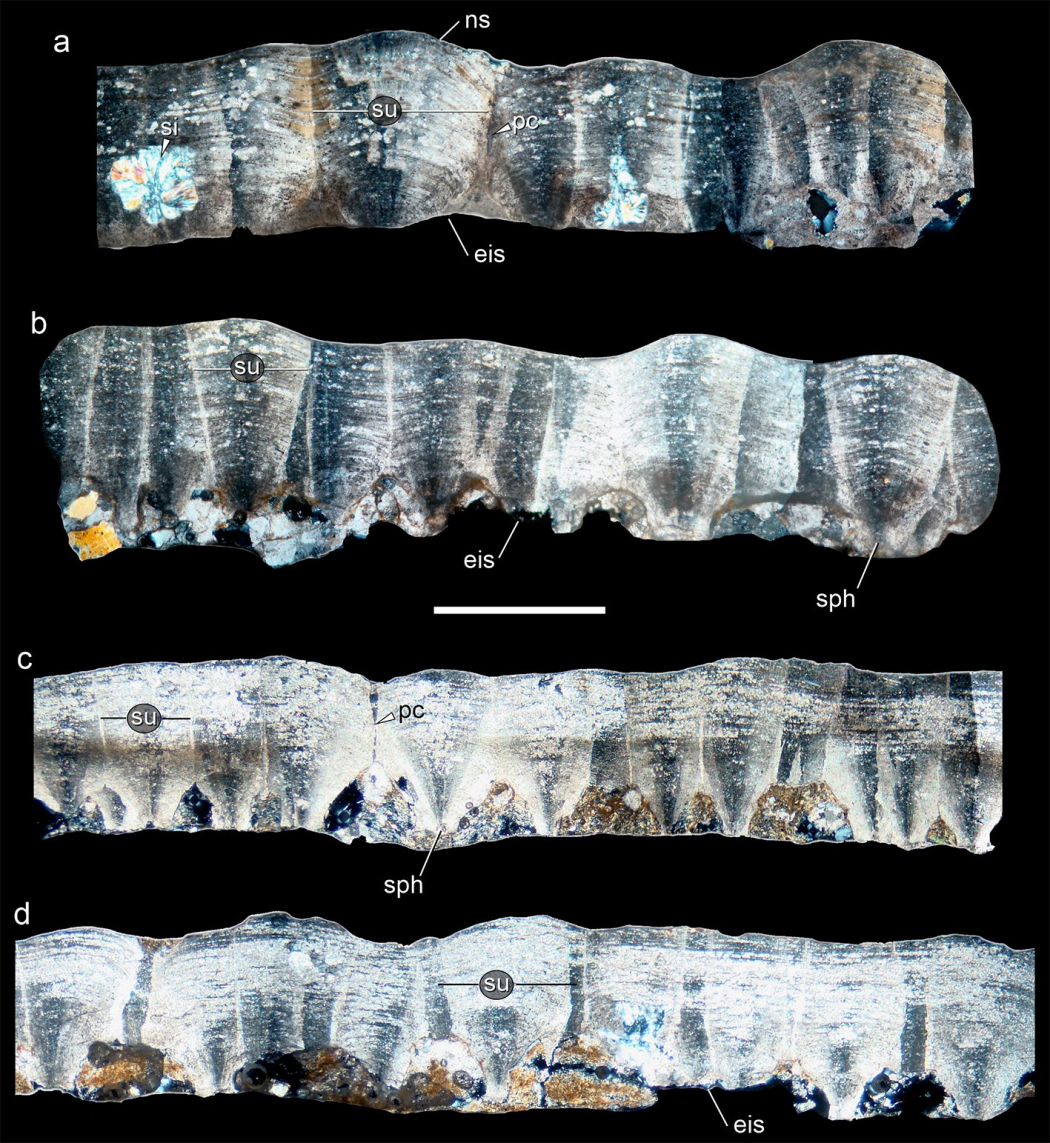
Transmitted light microscopic views of radial thin section of eggshell fragments from Ponte Alta nesting site showing the fan-shaped eggshell units. (**a**,**b**) Eggshells from CPPLIP 1798 clutch. (**c**,**d**) Eggshells from CPPLIP 1800 and 1801, respectively. The base of a group of eggshell units may develop “football boot-studs” shape. The matrix infilling of the eggs are represented by the sand and clayey matrix visible at the bottom of the eggshells. *eis* eroded internal surface, *ns* nodular surface, *pc* pore canal, *si* silicification, *sph* spherulite, *su* shell unit. Scale bar 1 mm.

**Figure 5:**
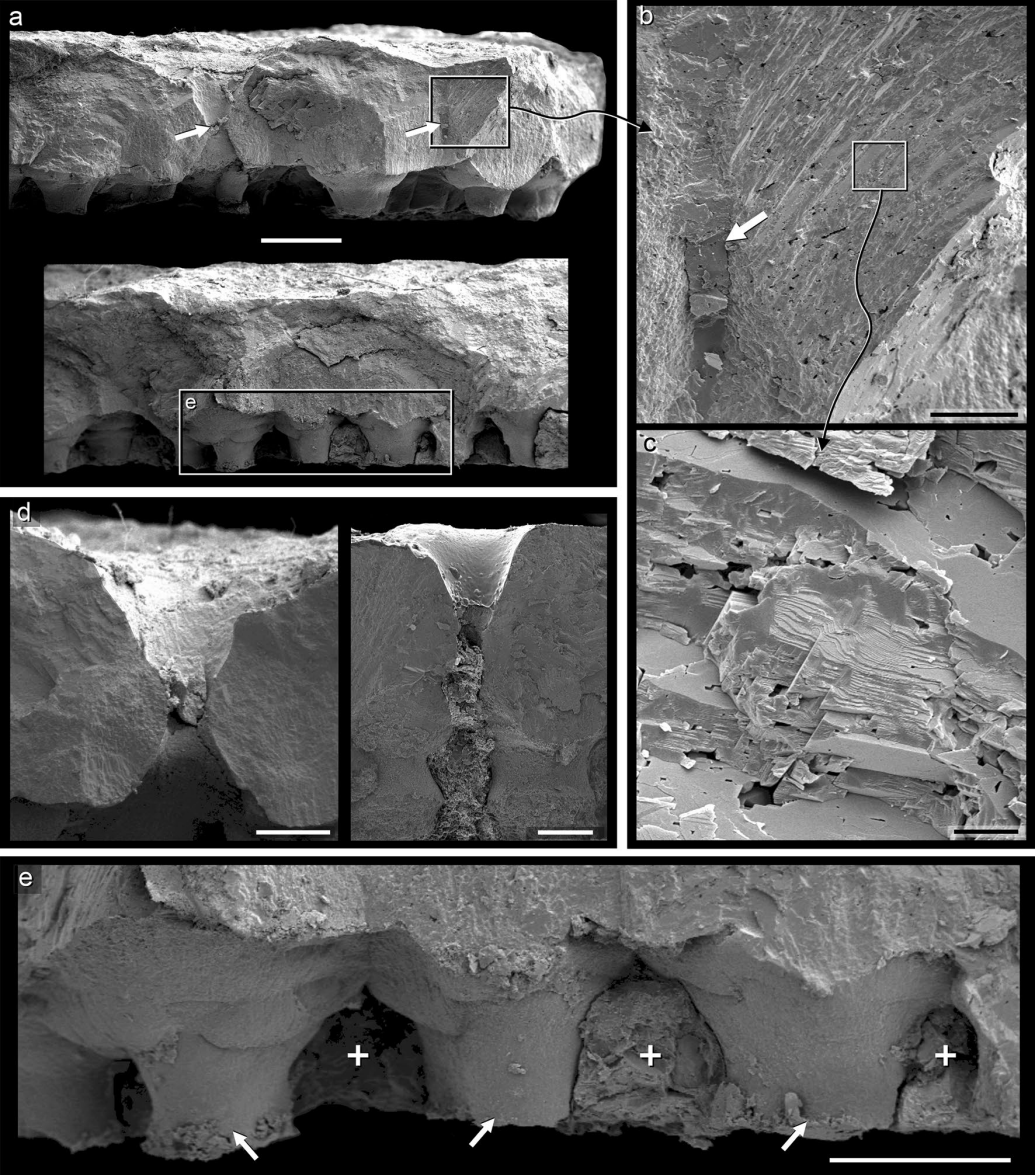
SEM images of eggshells from the Ponte Alta nesting site (taken from the clutch CPPLIP 1798). (**a**) Radial sections of pristine eggshells with some weathering outer (pore canal indicated by white arrows). (**b**) Eggshell growth of lines showing the pristine calcite of the unit with the infilling of the pore channel (white arrow) by secondary calcite deposits. (**c**) Original rhombohedric acicular calcite crystals forming and structuring the unit. (**d**) Very characteristic double funnel-shaped wide pores. (**e**) Well-developed base of eggshell units like “football boot studs” (white arrows) and the particularly evident network of horizontal pore canals running in between (white crosses). Scale bars represent 500 µm (**a**); 100 µm (**b**); 10 µm (**c**); 200 µm (**d**,**e**).

Despite these taphonomic alterations (mostly by pedogenesis), the eggs and their shells are generally very well preserved (Figs. 2, 4, 5). For those complete eggs with little deformations and breaks, we estimate an egg average diameter and volume of 12 cm and ~ 900 cm^3^, respectively (Fig. 2). These values are quite similar to other titanosaur eggs from Argentina and Romania^2,13,17,35^, although slightly smaller (~ 15 to ~ 20 cm) than other alleged titanosaur eggs distributed worldwide^15,17,19,20,36^. The specimen collected from an upper level, CPPLIP 1801, includes an almost complete and sub-spherical egg, with its major axis measuring 14.2 cm and a volume close to 1500 cm^3^. Computed tomography analyses revealed some important aspects of the eggs and clutches (Fig. 3). First of all, CT scans exposed the spatial distribution of eggs and no embryonic remains in ovo, a reliable way to accurately identify the taxonomy of a fossil egg^23^. In CPPLIP 1798 (Fig. 3b), some eggs have wide broken openings and contain eggshell fragments on the opposite side. In contrast, no high concentrations of shell fragments are evident outside the eggs, surrounding the clutch. Into the eggs, the eggshell fragments are found immerse in the sedimentary matrix, randomly with their concave face up or down (Fig. 3b–d). The lentil shape of the eggs suggests compaction and lithostatic pressure. Seen in plain view, its circular section is evident, but the shell is collapsed in the area of contact with two adjacent eggs (Fig. 3c,d).

The single structural layer of Ponte Alta eggshells (Figs. 4, 5) measures 1.1 mm in average and is thinner than other titanosaur eggshells. For example, the Tama eggshells averages 1.5 mm in thickness^20^; eggshells from Quebrada Santo Domingo, Auca Mahuevo, and Toteşti measure ~ 1.7 mm^2,13,35^; the mean thickness in Salitral de Bajo de Santa Rosa and south of France eggshells is ~ 1.9 mm^18,37^, whereas in Dholi Dungri and other nesting sites reach and exceed 2.5 mm^17,19,36,38^.

The eggshells are composed of densely packed fan-shaped discrete units (Fig. 4). These units vary between 350 and 600 µm in diameter and are structured by closely packed rhombohedral acicular calcite crystals radiating from nucleation centers at the base of each unit (Fig. 5). There is no substantial sign of recrystallization and pristine surfaces of small crystals of about 5 µm are observed (Fig. 5b,c). Although some show wear and erosion (Fig. 4), each unit exhibits the rounded convex top and collectively determines the profuse nodular external appearance of the eggshell surface. The combination of all these features (i.e., spherulites, nodular ornamentation, eggshell units shape, rhombohedral acicular calcite crystals, etc.) constitutes the typical design of titanosaur eggshells. The columnar base of each unit averages 250 µm in diameter (Fig. 5a,e); between each basal column, the complex network of the horizontal pore system occurs, well-developed in titanosaur eggshells. In general, the horizontal network is filled with sediment and there is no evidence of the fibrous membrana testacea beneath the units (Fig. 5e). Similar to the eggshell pores of Río Negro and Santo Domingo localities from Argentina^2,18,21^, straight and uniform pore canals of around 50 µm in diameter between the shell units are observed in the Ponte Alta specimens (Fig. 5b). However, display other kinds of pores with a peculiar and atypical morphology (Fig. 5d). Unlike the traditional eggshell pores found in other titanosaur eggs, some pores of Ponte Alta eggshells are very wide and have opposite and inverted double funnel-shaped, with large internal and external openings of around 350 µm and a medial bottleneck of around 100 µm (Fig. 5d). Moreover, the inner walls of these atypical pores show smooth pristine surfaces with no signs of internal erosion or recrystallization. These pores suggest a particular adaptability to the nesting microenvironment and their morphology could represent an apomorphic character for the titanosaur that nested in Ponte Alta site. In contrast to other titanosaur eggs, such those from Auca Mahuevo, Toteşti, and Tama^13,20,35^, the eggshell pores from this Brazilian nesting site do not ramify with a Y-shaped pattern.

## Discussion

The Ponte Alta nesting site is the first confirmed dinosaur nesting area for Brazil. No embryo remains have been yet recovered in ovo at Ponte Alta to effectively confirm and identify these eggs; however, the morphological features aforementioned of the eggs and eggshells perfectly match the eggs with titanosaur embryos found inside at other nesting localities worldwide^13,16,21,35,36^. The egg-clutches, egg-shape and size, and the eggshell morphology, e.g., the shape of units, the surface nodular ornamentation, and the vertical and horizontal pore network system (Figs. 3–5), suggest that the nests were made by titanosaurs and the occurrence of several clutches (Fig. 2) implies that in Ponte Alta there was a titanosaur nesting colony. During the Upper Cretaceous, between the Santonian to Maastrichtian, only derived titanosaurs such as saltasaurids and colossosaurians are recorded in South America^2,7^. However, only colossosaurian remains have been found so far in the Upper Cretaceous Bauru Group of Brazil^39–43^. This is highly suggestive that the Brazilian eggs, as with other Late Cretaceous embryonated eggs^16,21^, belong to some of these late titanosaur clades.

Isolated or fragmentary remains of titanosaur eggs had been previously reported for the Cretaceous of Brazil, even from the Ponte Alta region^28–31^. From isolated and fragmentary egg remains, Grellet-Tinner and Zaher^30^ already suggested that a group of titanosaur sauropods were present and reproducing in the region during the Late Cretaceous. Moreover, and because these animals would have had a generalized reproductive behaviour, these authors suggested colonial nesting for the Bauru Basin titanosaurs. The new Ponte Alta specimens support this hypothesis.

**Figure 6:**
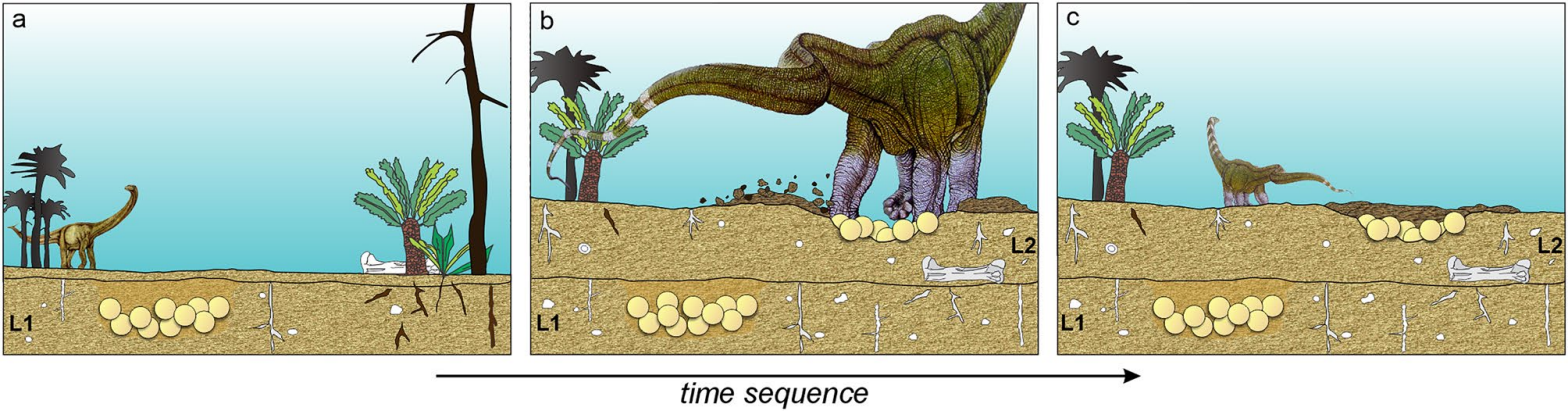
Model of events of titanosaur egg laying in two levels (L1 and L2), preservation, and subsequent sedimentation in the Ponte Alta nesting site. (**a**) First level of eggs. (**b**) Repeated selection of the laying area (by philopatry or breeding-site fidelity), excavation, and laying the eggs. (**c**) Covered eggs and a new deposition.

In addition to the colonial nesting behaviour, the finding of several eggs and egg-clutches accumulations in at least two levels suggests breeding philopatry (or breeding-site fidelity) (Fig. 6). This behaviour involves a group of individuals returning periodically to the same location to breed^44^ and can be selected for, particularly if local habitats are worth clinging to^45^. Besides, the population-level benefit of breeding-site fidelity in many cases requires finding better habitats, dispersion, and migration. Philopatry behaviour has been suggested for Cretaceous titanosaurs due to large and recurrent nesting breeding areas^2,13,16,19^. In this sense, it has also been suggested that many titanosaurs required specific conditions for nesting (e.g., at Sanagasta nesting site^11,19^) and would have migrated in search of these peculiar nesting areas. Philopatric behaviour, in addition to other specific intrinsic and extrinsic characteristics, further supports the idea that titanosaurs were breeding migrants^25^.

Titanosaurs are known to nest in a wide range of environments and varied nesting behaviours^11^. According to the fossil record, most species buried their eggs in the substrate to be incubated by solar or thermal heat, although some would have possibly explored mound-building strategies^11,15,19,20^. The egg-clutches of the Ponte Alta nesting site record two different levels within the palaeosol profile of the Serra da Galga Formation (Fig. 6), developed in Inceptisol palaeosols^32^. According to Soares et al.^32^, this Inceptisol palaeosol was formed in semiarid and well-drained palaeoenvironmental conditions. Similar characteristics of palaeosols and pedofeatures are seen in other titanosaur nesting sites (e.g., Tama nesting site)^20,46^ and suggest perhaps closely related titanosaur groups. Moreover, this evidence and morphological features of the eggs and eggshells advocate a nesting strategy similar to that displayed at Tama nesting site in La Rioja province, Argentina^20,46^. The palaeosol facies indicate long gaps in sedimentation of up to thousands of years generating topographic stability ideal for the nesting conditions of titanosaurs. Despite intense mining activity in Lafarge Quarry, the high density and co-occurrence of multiple egg-clutches in the same Inceptisol level bolster the hypothesis of a large breeding colony of dinosaurs in Ponte Alta (Fig. 6).

CPPLIP 1798 clutch, confirmed by CT-based 3D reconstruction, shows a compact arrangement of the sub-spherical eggs in two overlapping layers, which suggests in situ preservation and incubation within the sandy substrate. CPPLIP 1798 is the most informative in terms of spatial distribution, with several eggs overlapping and contacting each other. The arrangement in the lower layer, with central eggs located lower than those on the periphery of the accumulation, is consistent with deposition in a bowl-shaped structure. This is a common feature in titanosaur clutches and its nesting behavior due to buried nest building^11,15,19,20,23^. In addition, most eggs show signs compatible with vertical compression due to sedimentary loading, such as the lentil shape and fractures in one of the egg halves. Several eggs with wide openings could be also considered signs of biological activity, either hatching or scavenging. However, these activities generally displace debris both inside and outside the egg. In CPPLIP 1798 the missing fragments appear exclusively within each egg. Furthermore, numerous isolated eggshells are immersed in the matrix, which suggests that they were incorporated as the sediment entered the egg during diagenesis. These patterns suggest that the eggs fractured after the matrix lithified suffering lateral extension^47^. This proves that the eggs were complete at burial and titanosaurs from Ponte Alta buried their eggs. The compaction by sedimentary load is also the most plausible hypothesis to explain the disposition of the broken egg, whose debris is similar to that of another egg located further down.

Although it looks very similar to other titanosaur eggshells (e.g., Tama, Quebrada Santo Domingo, Auca Mahuevo, or Toteşti), the thin shell of the Ponte Alta eggs displays some atypical pore canals. In addition to the typical fine and straight pores with funnel-shaped openings, they display wide interspersed and apparently randomly distributed pores. These pores show wide internal and external funnel-shaped openings up to 350 µm but no forked Y-shaped pattern like in Auca Mahuevo, Toteşti, and Tama eggshells^13,20,35^. The pore canals facilitate gas exchange through the eggshell and the supply of oxygen necessary for the embryo^48^. The pore canal morphology and the characteristics of this complex respiratory system determine the gas conductance and the humidity in the surrounding nesting environment^49^. Actually, it is the most informative trait of the eggshell and their micro-nesting features^20^. The atypical wide opposite and inverted double funnel-shaped pores observed in the Ponte Alta eggshells display pristine inner wall surfaces (Fig. 5d). These pores are not the product of dissolution, erosion, recrystallization or any other taphonomic artefact, but their specific shape. These peculiar wide pores would have favored enormous gas diffusion through the thin eggshell, while the constriction in the midsection—like a bottleneck—would have restricted this diffusion. Thus, these pores increase the pore area and could be a physiological response in those cases of pores clogged by sand grains allowing a continuous exchange of gases and maintaining a high diffusion. Moreover, this suggests an adaptive response to reproduction under relatively arid environmental conditions, a very common behaviour in mid-palaeolatitude titanosaurs^25^.

Regarding to the palaeolatitude, during the Late Cretaceous, Ponte Alta was located at about -26 degree (similar to the Indian nesting sites), but lower than the other nesting sites in South America (between -33 to -47 degrees). This latitudinal difference could also influence the distribution of species on the Gondwana continent, and the palaeoclimatic variation and geological characteristics could be determinant at the time of nesting. Cretaceous titanosaurs once roamed over all continents, even walked in Antarctica. Their skeletal remains were found everywhere^1–6^, whereas titanosaur nesting sites have more restricted distributions, being recovered in Spain, France, Rumania (Laurasia) and Argentina, India, and now Brazil (Gondwana). However, the presence of isolated eggs or eggshells in other areas (e.g., Peru, Uruguay)^26,27^ suggests geographically broader nesting areas yet to be discovered. Tightly packed nesting colonies were seemingly very common among titanosaurs; some South American colonies would have been really huge (e.g., Auca Mahuevo, Sanagasta, Santo Domingo, etc.), establishing true titanosaur “rookery”. However, these rookeries developed with a strong dependency on a wide variety of environments and exploited diverse nesting behaviors^11^, which would have been a tremendous adaptive success for the group.

## Conclusions

We report the first dinosaur nesting area for the Cretaceous of Brazil, corresponding to several eggs and egg-clusters discovered in the Serra da Galga Formation (Bauru Group) at the abandoned mining Lafarge Quarry, in Ponte Alta region, Uberaba Municipality, Minas Gerais State. This titanosaur nesting site also represents the most boreal one for South America, with a palaeolatitude similar to the one found in India. Fossil preparation and CT scans of these egg-clutches have so far not revealed any embryonic remains in ovo. However, the egg-clutch features and the macro- and micromorphology of the eggs and their eggshells match those titanosaur eggs found worldwide which bolster the hypothesis of Late Cretaceous derived titanosaurs nesting in large colonial breeding areas also in Brazil. Based on the depositional horizon and the macro- and micromorphology of the eggs, the titanosaurs from Ponte Alta must have adopted a burial nesting strategy and the eggs were incubated in specific conditions under environmental source heat. These would have been commonly chosen nesting conditions by lithostrotian titanosaurs. During Cretaceous times, titanosaurs lived on every continent, even Antarctica. The worldwide evolutionary success of titanosaurs was due, among other things, to their great, quasi-general, adaptive behaviour to nest in colonial nesting areas and in several environments. However, the direct dependence of its nesting behaviour to specific environments (e.g., arid palaeosols, hydrothermal environment, etc.) could have played a key role as an extinction factor at the end of the Cretaceous.

## Methods

### Institutional abbreviations

CPPLIP, Centro de Pesquisas Paleontológicas “Llewellyn Ivor Price”, Complexo Cultural e Cientifico de Peirópolis, Universidade Federal do Triângulo Mineiro, Peirópolis, Uberaba, Minas Gerais, Brazil. All the egg-clutches, eggs, and eggshell fragments, petrographic samples and thin-sections here studied are housed at the CPPLIP.

### Sample collection

Our data consist of titanosaur egg-clutches, eggs, and eggshell specimens collected between 1998–2000 during mining work for the extraction of limestone in the now abandoned Lafarge Quarry (Ponte Alta, Minas Gerais State, Brazil), where the Serra da Galga Formation is widely exposed^32^. The recovered specimens were donated and incorporated into the CPPLIP collection through the management carried out by two of the authors (JIDS and AGM). The exact point where the egg-clutches were found at the Lafarge Quarry is here nominated “Ponte Alta nesting site” (GPS: S19° 42.634′/W47° 40.534′, 844 m.a.s.l.). Several eggshell fragments were selected for microscopy, on which were made thin sections for petrographic analyses as well as coated with metal for scanning electron microscope (SEM) analyses and images. Comparisons with other eggs and eggshells of titanosaurs were made on the basis of descriptions and figures in the literature as well as from egg material studied first-hand.

### Eggshell thin sections

The thin sections of eggshell fragments were made at the Petrology Laboratory at CRILAR (La Rioja, Argentina), using the following protocol for petrographic sectioning: the specimens were washed in distilled water and cut with PetroThin, dried at 40 °C in an oven for 24 h, glued with compound glue (Araldit CY 248 and hardening HY 956) on glass slides of 28 × 48 × 1.8 mm^3^. Observations were obtained under a Leica DM2500P petrographic microscope and images were captured with a digital camera (Leica DFC295) attached to the microscope and connected to a computer for processing, editing, and measurements.

### SEM analysis

The scanning electron microscopic analysis and preparation of the eggshell materials and image captures were performed following^50^. Each specimen was mounted on an aluminium stub and coated with gold/palladium for 15 min (3 Armstrong/seg.) in a Thermo VG Scientific SC 7620. The SEM observations were conducted at 10 kV with a LEO 1450VP Scanning Electron Microscope at the Microanalysis Laboratory of the Universidad Nacional de San Luis (San Luis, Argentina; http://labmem.unsl.edu.ar/).

### CT analysis

Specimens CPPLIP 1798, CPPLIP 1799 and CPPLIP 1801 were scanned at the Toshiba Aquilion^®^64 helical CT scanner of the Centro de Diagnóstico por Imagen, Hospital de Clínicas of the Universidade Federal do Triângulo Mineiro (UFTM, Uberaba, Minas Gerais State, Brazil). CPPLIP 1798 has 512 images, CPPLIP 1799 has 375 and CPPLIP 1801 has 157. The slices have 1 mm of thickness, a voxel size of 938 μm, 608 μm and 361 μm, respectively, and were done under 120 kVp and 200 μA. The three-dimensional reconstructions were performed by using the open source software 3D Slicer.

### Image and graphics design

CorelDRAW Graphics Suite 2020 and Adobe Photoshop CC14 were used for storage and processing of the images, and final design of the figures. All photos and drawings of the figures were taken and drawn by the authors (LEF, AGM, EMH, and MVTS).

### On the methodology of egg parataxonomy

Herein we do not follow the classic oologic parataxonomic methodology and classification^51^ because it presents serious biological-evolutionary problems. We introduce here some of them. Although parataxonomy is extensively used in studies of fossil eggs, we analyzed the Ponte Alta eggs as a biological entity within the context of the evolutionary trend of amniotes. After fertilization of an egg cell, the eggshell—and membranes—is an organic complex structure containing genetic information of the female and combined genetic information in the embryo derived from the sexual reproduction (unless parthenogenesis has occurred). Unlike the embryo, the rest of the egg (shell and membranes) is genetically determined by the female. In general, the dinoavian eggshell is about 95% calcium-carbonate mineral—calcite—and ~ 3.5% organic material/matrix^52^ (unlike bone tissues, which are generally made up of calcium phosphate and organic materials in almost similar proportions). For that reason, the eggshell is a biocomposite ceramic consisting of organic matrix—hundreds of identified proteins—and a crystalline calcium carbonate filler^53^. The large number of proteins that make up the shell (e.g., ovocalyxin, lysozyme, ovalbumin, ovocleidin, cystatin, osteopontin, ovotransferin, X-type collagen, keratan and dermatan sulfate, etc.) determine the processes of deposition or inhibition of calcite—shell crystallization and pore, respectively^54,55^. In this sense, the eggshell is a biomineralized structure which occurs in the uterus, and is the product of complex physiological processes that depend on deep and precise hormonally and genetically synchronized moderators^52,54–58^. Like bone tissue, the dinosaur eggshell tissue is a bioceramic with phylogenetic information and provides multiple functions to the embryo^23,57,58^. There are at least four ways to determine the taxonomy of a fossil egg: (1) with an embryo inside^13,16,21,22,59–61^; (2) adults with eggs inside^62^; (3) adults brooding eggs^63,64^ and (4) by phylogenetic inferences from the egg and its shell^23,50,60^. In most egg and eggshell fossil cases should be approached from this last perspective. Thus, the eggs described here certainly correspond to a specific dinosaur clade within Sauropoda and their oological and reproductive features can be phylogenetically traceable^23^. And under the light of evolutionary biology, fossil egg parataxonomy classification is inappropriate. For these and other reasons, we do not use it here.
